# Supplementary material for: Identifying Behavioural Determinants to Uptake and Adherence to a Whey Protein Supplement for the Management of Type 2 Diabetes: A Qualitative Interview Study
Source: Nutrients. 2022 Jan 27;14(3):565. doi: 10.3390/nu14030565 (PMC8840711; doi:10.3390/nu14030565)
Supplement: Supplementary file 1 [file nutrients-14-00565-s001.zip › nutrients-1493157-supplementary.pdf]

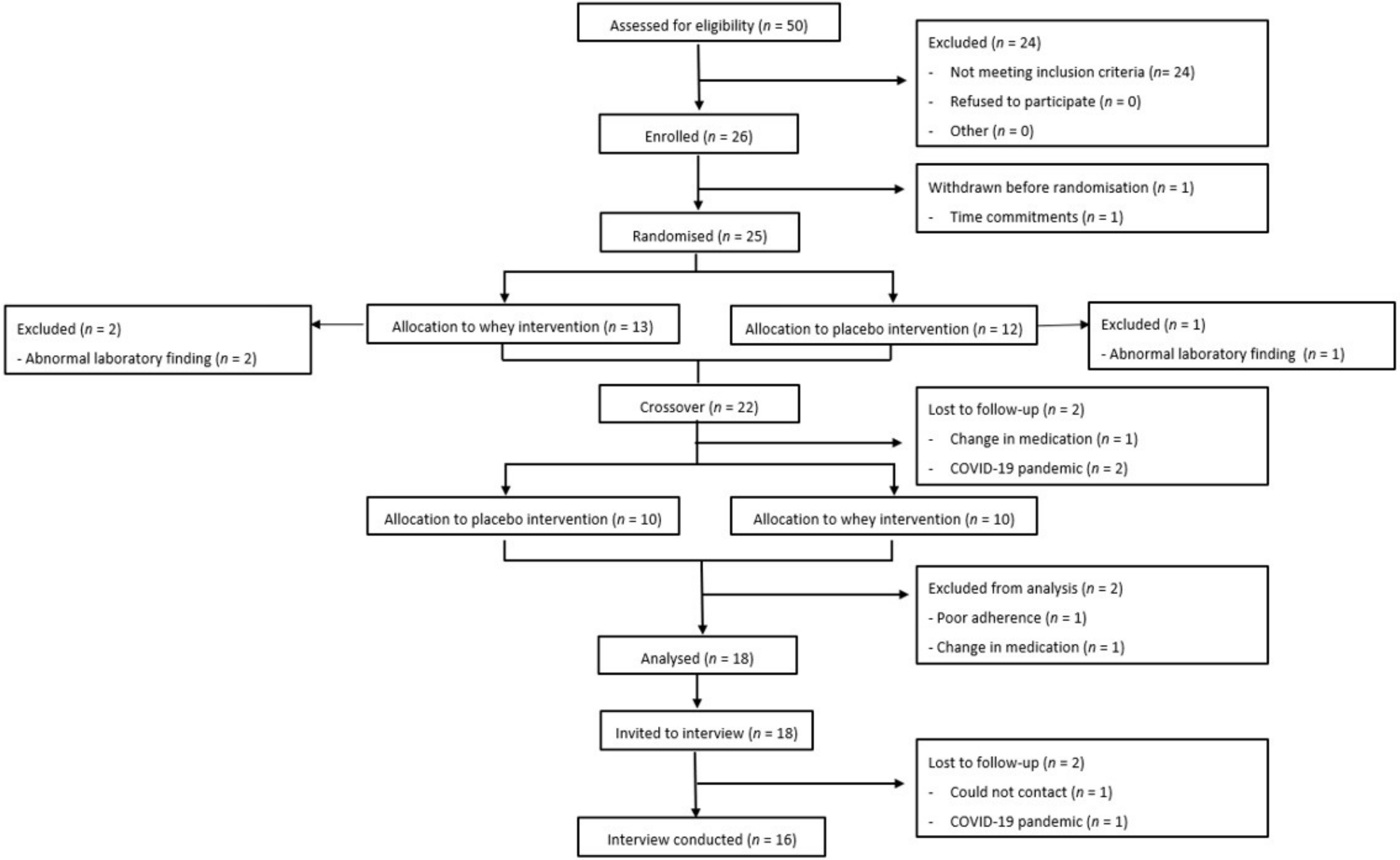

Figure S1. Semi-structured interview topic guide

The Newcastle upon Tyne Hospitals NHS
NHS Foundation Trust
Royal Victoria Infirmary
Queen Victoria Road
Newcastle upon Tyne
NE1 4LP
Tel: 0191 233 6161
Fax: 0191 2010155
www.newcastle-hospitals.nhs.uk

Newcastle University

Topic Guide

1)	What were your reasons for taking part in this study?<br><br>**Prompts:** To improve my diabetes; to prevent diabetes complications; financial incentive; interested in the findings of the research in general; interested in the findings of the research for myself; I have tried other things that haven't worked; my diabetes has got progressively worse over the years; I have become more motivated over time to better manage my diabetes
2)	a) What have you tried in the past to manage your diabetes?<br><br>**Prompts:** lifestyle changes; medications; taken part in other studies of novel therapies<br><br>b) if yes, did this work for you?<br><br>**Prompts:** what did you achieve with it?<br><br>c) Do you still continue with this/these approaches?<br><br>**Prompts:** why if not very successful? What do you feel are the specific benefits for you?<br><br>d) How difficult is it to stick to this approach?<br><br>**Prompts:** what are the obstacles?
3)	To what extent do you believe your diabetes will get worse if you continue with your current management approach?
4)	Before you started this study, to what extent did you believe this new supplement would improve your diabetes?<br><br>**Prompts:** What were your expectations and why?
5)	How quickly did you expect the supplement to have benefits on your health and well-being?<br><br>**Prompts:** blood glucose control; sense of well-being; feelings of taking control
6)	Now that you have used the supplement could you tell me whether it met your expectations in terms of the results?<br><br>**Prompts:** Results generated, ease of use
7)	a) Thinking about taking the supplement every day before each meal, how did you find this?<br><br>**Prompts:** As expected; difficult to remember [I sometimes forgot]<br><br>b) How did it fit with your daily routine?

8)	Overall, how did you find the supplement?<br><br>**Prompts:** taste; texture; ease of use
9)	Did the supplement have any effect on your appetite or eating patterns? If so, could you explain further?
10)	How did you feel at various times of the day while taking the supplement, for example, was it easier to take in the morning versus the afternoon?
11)	Were there any differences in how you felt between taking the supplement the first time around compared to the second?<br><br>**Prompts:** Were the feelings described earlier the same or different
12)	a) What did you like about taking the supplement?<br><br>b) What did you not like about taking the supplement?
13)	a) : To what extent do you believe you could carry on taking this supplement as part of your daily routine?<br><br>b) What might be the challenges?<br><br>c) What would increase the likelihood that you would continue to take this supplement for your diabetic care? i.e., ingestion closer to or further before meals? Having a larger shot less frequently (i.e., once or twice daily)?
14)	a) What did you find most challenging about taking the supplement?<br><br>b) What kind of things did you do to make taking the supplement easier?<br><br>**Prompts:** Set reminders so didn’t forget to take it; changed meal times; mixed it with food/drink
15)	Would you recommend this supplement to other people with type 2 diabetes?<br><br>**Prompts:** Yes/No why?
